# Globally sclerotic glomeruli in IgA nephropathy patients

**DOI:** 10.12861/jrip.2012.03

**Published:** 2012-01-01

**Authors:** Peyman Mohammadi Torbati

**Affiliations:** ^1^Department of Pathology, Shahid Beheshti University of Medical Sciences, Tehran, Iran

**Keywords:** Immunoglobulin A nephropathy, Oxford classification, Sclerotic glomeruli

**Implication for health policy/practice/research/medical education:**

Oxford classification system was based on actual clinical data, international collaboration, and validation of the reproducibility of defining pathologic lesions. The strongest pathologic predictor of clinical outcome in IgA nephropathy is the extent of tubular atrophy and interstitial fibrosis.

IgA nephropathy is the most common glomerulonephritis worldwide (1). Over the years, several pathologic classifications have been devised to determine prognosis in individual patients, but none has gained widespread acceptance. In Berger's original cohort of IgA nephropathy patients, progression to end-stage renal disease (ESRD) was rare, and throughout the 1970s and 1980s IgA nephropathy was generally felt to be a benign disease, supported by studies from Europe, Asia, and Australia indicating actuarial 10-year renal survival rates from the time of diagnosis or renal biopsy of greater than 80% (1-3). Over the past 15 to 20 years, however, it has become evident that IgA nephropathy is not as benign as once thought (1,2). It is now generally felt that most patients with IgA nephropathy develop a progressive decline in renal function, though at a highly variable rate, and 15% to 40% of patients will eventually develop ESRD (1). A well-defined pathology grading system must contribute additional prognostic information beyond that provided by clinical features. It seems that the Oxford classification system for IgA nephropathy provides this criterion. This system was based on actual clinical data, international collaboration, and validation of the reproducibility of defining pathologic lesions. In the original study of 265 patients published by the Oxford Group, four lesions including: endocapillary hypercellularity, mesangial hypercellularity, segmental sclerosis, and tubular atrophy/interstitial fibrosis (IF/TA), were found to predict clinical outcome (1-3). Surprisingly necrosis, crescent formation was not identified as prognostic factors in Oxford study (4). Finally, the significance of global glomerular sclerosis was not informative (1,2). The strongest pathologic predictor of clinical outcome in IgA nephropathy is the extent of tubular atrophy and interstitial fibrosis (1,2). In nearly all studies in which this has been examined, tubulointerstitial scarring is an independent predictor of progression to ESRD in a multivariate analysis including various pathologic and clinical parameters. By contrast, although glomerular sclerosis has been found to correlate significantly with disease progression by univariate analysis in nearly all studies, this is often not an independent predictor of such progression by multivariate analysis (1-4). In recent study, Nasri *et al*. examined the effect of global glomerular sclerosis on a cohort of 136 IgAN patients from Isfahan, Iran. They showed a significant positive correlation between proportion of globally sclerotic glomeruli and serum creatinine, amount of proteinuria and also quantity of IF/TA (5). It seems that assessment of significance of global glomerular scar in relation to annual measurement of glomerular filtration rate (GFR) in a period of least 5 years may provide more realistic results. Yet the main questionable issue is about the significance of glomerular scar as an independent predictor of clinical outcome. Nasri *et al*. conducted a multivariate analysis for a small group of IgAN patients and they found that the best correlation can be established between global glomerular scar and tubulointerstitial fibrosis (5). Also there is a good correlation between segmental and global scar with clinical parameters. Confounding data which are gathered from univariate and multivariate analysis highlight the need for large prospective studies to better define the contribution of pathology lesions to clinical outcomes.

## Author’s contribution

PMT is the single author of the manuscript.

## Conflict of interests

The author declared no competing interests.

## Ethical considerations

Ethical issues (including plagiarism, data fabrication, double publication) have been completely observed by the author.

## Funding/Support

None.
